# Late presenters to HIV care and treatment, identification of associated risk factors in HIV-1 infected Indian population

**DOI:** 10.1186/1471-2458-10-416

**Published:** 2010-07-13

**Authors:** Kamalika Mojumdar, Madhu Vajpayee, Neeraj K Chauhan, Sanjay Mendiratta

**Affiliations:** 1 Department of Microbiology, All India Institute of Medical Sciences, New Delhi, India

## Abstract

**Background:**

Timely access to antiretroviral therapy is a key to controlling HIV infection. Late diagnosis and presentation to care diminish the benefits of antiretrovirals and increase risk of transmission. We aimed to identify late presenters in patients sent for first CD4 T cell count after HIV diagnosis, for therapy initiation evaluation. Further we aimed at identifying patient factors associated with higher risk of late presentation.

**Methods:**

Retrospective data collection and analysis was done for 3680 subjects visiting the laboratory for CD4 T cell counts between 2001 and 2007. We segregated the patients on basis of their CD4 T cell counts after first HIV diagnosis. Factors associated with risk of late presentation to CD4 T cell counts after HIV diagnosis were identified using univariate analysis, and the strength of association of individual factor was assessed by calculation of odds ratios.

**Results:**

Of 3680 subjects, 2936 (83.37%) were defined as late presenters. Late testing varied among age groups, transmission categories, and gender. Males were twice as likely to present late as compared to females. We found significant positive association of heterosexual transmission route (*p *< 0.001), and older age groups of 45 years and above (*p *= 0.0004) to late presentation. Female sex, children below 14 years of age and sexual contact with HIV positive spouse were associated with significantly lower risks to presenting late. Intravenous drug users were also associated with lower risks of late presentation, in comparison to heterosexual transmission route.

**Conclusions:**

The study identifies HIV infected population groups at a higher risk of late presentation to care and treatment. The risk factors identified to be associated with late presentation should be utilised in formulating targeted public health interventions in order to improve early HIV diagnosis.

## Background

AIDS is a dreaded disease, as even with recent advances in the field of medicine and public health, no effective vaccine or drug therapy is available to completely eliminate the infection. During the natural course of HIV infection, there is a progressive loss of CD4 T cells; the rate of this loss being variable in patients, but averaging around 60-100 cells/uL per year [1-3]. This drop in CD4 T cells leads to a severely immunocompromised state in the infected host. Thereupon, a reduction in CD4 T cells to below 200 cells/uL makes the host highly susceptible to opportunistic infections and increases overall AIDS related morbidity and mortality. It is universally documented that in the absence of effective antiretroviral therapy, most people infected with HIV will progress to AIDS in approximately ten years, with this period varying from patient to patient based on host and viral factors [4].

Introduction of highly effective antiretroviral therapy (HAART) has substantially improved patient prognosis over the past decade [5,6]. Testing, diagnosis, and medical care soon after HIV infection and before developing opportunistic infections and other AIDS defining illness and clinical AIDS, can prevent illness, improve survival, and reduce transmission. However, patients receiving HIV diagnosis late in the course of infection are usually more severely immunocompromised and are more likely to present with co-morbidities like tuberculosis, and have short-term mortality [7]. Delay in diagnosis is significant to both disease prognosis at patient level and transmission at community and public health level. An early diagnosis provides opportunities of reducing or halting further transmission due to changes in risk behaviour [8]. Further, due to a higher viral burden in these patients, the likelihood of transmission from these patients is also very high compared to individuals diagnosed early in the course of infection. Early diagnosis and immediate initiation of therapy are essential components for the success of HIV control and prevention programs.

Recent data indicates that early initiation of HAART is advantageous when opportunistic infections are present [9], as the overall mortality is excessively high in patients with very late HIV disease and opportunistic infections. Delay in initiating therapy in these patients is associated with increased risk of mortality. Also, Immune Reconstitution Inflammatory Syndrome (IRIS) which is associated with low CD4 T cell counts at baseline occurs at a higher frequency in subjects with late HIV diagnosis [10]. The present study was carried out at one of the nation's biggest tertiary care hospital, the All India Institute of Medical Sciences (AIIMS) located in Delhi, the national capital. The hospital caters to patients basically from Delhi and the surrounding national capital region, which includes Punjab, Chandigarh, Uttar Pradesh, Uttaranchal, Uttarakhand, etc. However, it being the nation's premier medical institute, we see influx of patients from most states of India. In the present study, we aimed at assessing the immunological profile of HIV infected Indian patients at the time of first CD4 T cell count testing, and to identify late presenters based on these counts. Also, we examined proportion of subjects with higher CD4 T cell counts, but with clinical complications related to HIV disease, and thus being in immediate need of HAART initiation. Further, we try to identify factors which make an individual more susceptible to being a late presenter to HIV diagnosis and care.

## Methods

### The HIV scenario

India, with 2.27 million people living with HIV/AIDS and a disease prevalence of 0.29% (2008-09), accounts for roughly half of Asia's HIV prevalence. New Delhi, the area where the study was conducted, is a low prevalence state. However, being the nation's capital, the state sees a huge influx of immigrants and truckers, thereby increasing the high risk population. Most of the infections occur through the heterosexual route, though the major transmission route in north-eastern India is injection drug use. The first phase National AIDS Control Programme (NACP) was launched in 1992 with the objective of slowing down the HIV infections in order to reduce the morbidity, mortality and impact of HIV epidemic in the country. Since then, the third phase of NACP has been planned and implemented in 2007 with the objective of halting and reversing the HIV epidemic in India over a period of five years. The guiding principle of the NACP - III objectives is provision of universal access to HIV prevention, care, and support and treatment services. The current HIV testing guidelines states client initiated testing, except in case of pregnant females in which case HIV testing is provider initiated. HIV treatment and monitoring is universally accessible and free of cost at NACO designated clinics, and one such clinic is housed in the AIIMS, which is the study's focus. The institute's HIV division consists of an Integrated Counselling and Testing Centre, in addition to a CD4 monitoring laboratory and treatment clinic, all funded and supported by the national program and NACO. The CD4 monitoring laboratory at AIIMS, is housed in the Microbiology department, and provides the monitoring services to all patients referred to it through various OPDs, wards, and specialty clinics at AIIMS providing HIV treatment and care.

### Study subjects

This is a retrospective cross sectional study of all individuals coming for CD4 testing for the first time after HIV diagnosis (usually within a week of positive HIV serodiagnosis), to the CD4 testing laboratory at the Department of Microbiology, All India Institute of Medical Sciences. Subjects included 3,680 HAART naïve individuals with a confirmed diagnosis of HIV with or without clinical symptoms, coming to the laboratory between 2001 and 2007. Subjects on antiretroviral therapy at the time of first visit to the laboratory were excluded from the present study analysis. Similarly, subjects with a positive HIV diagnosis more than one month before entering the HIV care services at AIIMS were also excluded from the study in order to control for the time since first HIV diagnosis. The All India Institute of Medical Sciences Institute Ethics Committee (AIIMS IEC) approved routine HIV diagnosis and CD4 testing.

### Criteria for categorization as "late presenters to care"

For data analysis, as made classically all subjects with CD4 T+ cell count below 200 cells/uL were classified as 'late presenters to care". Subjects were also stratified into late and very late presenters depending on their CD4 T cell counts being below 200 cells/uL and below 50 cells/uL respectively. Further, subjects with CD4 T cell counts above 200 cells/uL but with chronic clinical symptoms like fever, diarrhoea, weight loss etc, were also included in the category of late presenters to care and in need of immediate start of HAART.

### Data collection

Laboratory CD4 test subject database was accessed to obtain following patient characteristics: age, sex, date of CD4 testing, HIV transmission route as reported by the patient, and clinical classification of patients on presence or absence of symptoms. Presence of TB, both pulmonary and extrapulmonary, and Sexually Transmitted Diseases (STDs) were also recorded.

### Statistical analysis

Medians were calculated for study variable: age, CD4 T cell counts, etc. Univariate regression analysis was performed to identify patient factors which varied among late presenters and non-late presenters. Multinomial logistic regression analysis was performed to identify independently associated patient factors with risk of presenting late for HIV related treatment and care. Confidence intervals at 95% level of confidence were calculated for each associated factor. A p value of 0.05 or less was considered significant.

## Results

During 2001-2007, a total of 4,260 patients were tested for CD4 T cell counts at the laboratory. Of these subjects, 3,680 subjects coming for HAART initiation evaluation were retrospectively included in the study, excluding subjects coming for follow-up visit or subjects already on antiretroviral therapy at the time of first visit to laboratory. Patient characteristics according to CD4 T cell counts is summarized in Table 1.The study subjects were primarily males (2,598, 70.6%) aged between 25-44 years (2,661, 72.36%) reporting heterosexual promiscuity as the mode of HIV acquisition (1,475, 40.1%).

**Table 1:** Study subject characteristics

Characteristics	Number	Percentage (%)
**Year of testing**		

2001	275	7.5

2002	495	13.4

2003	457	12.4

2004	612	16.6

2005	535	14.5

2006	767	20.8

2007	539	14.6

**Sex**		

Male	2598	70.6

Female	1082	29.4

**Age Group**		

0-14 years	198	5.4

15-24 years	398	10.8

25-34 years	1612	43.8

35-44 years	1049	28.5

45 years & above	423	11.5

**Transmission route**		

Heterosexual	1475	40.1

HIV+ spouse	783	21.3

Parent to child	133	3.6

Transfusion recipient	428	11.6

IVDU	43	1.2

Unsterile syringe use, Occupational exposure	32	0.9

MSM	4	0.1

Unknown, undisclosed	782	21.2

**Symptoms**		

Asymptomatic	745	20.2

Symptomatic	2935	79.8

**AIDS related/defining illnesses**		

TB	529	14.4

Sexually Transmitted Infections	102	2.8

Other AIDS defining illnesses	564	15.3

Majority of subjects presented with one or more AIDS related and/or AIDS defining illnesses [11,12] at the time of first CD4 T cell count (3,068, 83.4%). Among these, 529 (14.4% of total) subjects had pulmonary or extrapulmonary TB, 102 (2.8%) had sexually transmitted infections at the time of testing, and 564 subjects (15.3%) had at least one AIDS defining illness.

The study group had a median CD4 T cell count of 242 cells/uL. Males had a lower median CD4 T cell count (207 cells/uL) as compared to females (243 cells/uL) with this difference being statistically significant (p < 0.001). Of the total study population, 2,113 (57.4%) had CD4 T cell counts more than 200 cells/uL, 1,214 (33%) had CD4 T cell count below 200 cells/uL, and 353 (9.6%) had less than 50 CD4 T cells/uL. Patient characteristics according to CD4 T cell counts are summarised in Table 2. Six hundred and twelve (612) subjects with CD4 T cell counts greater than 200 cells/uL, 120 subjects with less than 200 cells/uL, and 13 subjects with less than 50 CD4 T cells/uL were asymptomatic at the time of CD4 test.

**Table 2:** Distribution of study subject characteristics based on CD4 T cell counts

Characteristics	CD4 T cell counts (cells/uL)			Overall p value
		
	> 200	< 200	< 50	
**Sex**				

Male	1338	957	303	**< 0.001**

Female	775	257	50	

**Age Group**				

0-14 years	158	33	6	

15-24 years	307	75	17	

25-34 years	925	541	146	**< 0.001**

35-44 years	504	404	141	

45 years & above	219	161	43	

**Transmission route**				

Heterosexual	779	535	165	

HIV+ spouse	605	158	20	

Parent to child	112	18	3	

Transfusion recipient	212	167	49	**< 0.001**

IVDU	30	11	2	

Unsterile syringe use, Occupational exposure	11	17	4	

MSM	1	2	1	

Unknown, undisclosed	363	310	109	

**Symptoms**				

Asymptomatic	612	120	13	**< 0.001**

Symptomatic	1509	1086	340	

Subjects with less than 200 CD4 T cells/uL with (n = 1086) or without clinical symptoms (n = 120), and subjects with clinical HIV infection irrespective of CD4 T cell counts (1509), were together termed as "late presenters to care". Further, subjects with less than 50 CD4 T cells/uL were termed "very late presenters", of which 340 subjects were symptomatic and 13 subjects did not have any clinical symptoms. Four hundred and seventy three (473) late presenters (17.4% of 2723 late presenters) and 91 very late presenters (26.5%, of 343 very late presenters) had clinically defined AIDS at the time of first presenting to the laboratory.

Univariate analysis was performed for association between patient characteristics and late presentation, summarised in Table 2. Significant differences in proportion of late presenters were recorded among subjects reporting different modes of disease acquisition, with highest late presentation seen in subjects reporting heterosexual promiscuity. Frequency of late presenters was highest in males and age group 25-34 years, with all above differences in proportion being statistically significant (p < 0.001).

Multinomial logistic regression analysis was carried out to identify patient factors independently associated with late and very late presentation to care. In this analysis we found that females were at a significantly lower risk to present late or very late for CD4 testing as compared to their male counterparts (late presentation: OR 0.49; 95% CI 0.41 - 0.54, very late presentation: OR0.21; 95% CI 0.15 - 0.30). Similarly age groups 35-44 years (late presentation: OR 1.72; 95% CI 1.39 - 2.12, very late presentation: OR 2.78; 95% CI 2.07 - 3.72), and age group of 45 years and above (late presentation: OR 1.77; 95% CI 1.28 - 2.44, very late presentation: OR 1.76; 95% CI 1.13 - 2.74) were associated with higher frequency of late presentation.

On analysing various transmission routes reported by subjects for their association with propensity for late presentation to care, transfusion of blood and blood products, homosexual transmission, occupational exposure and those unaware or unwilling to disclose probable route of HIV acquisition, were all found to increase the risk of late presentation. Further we observed that female sex, age at presentation less than 25, mother to child route of transmission, HIV transmission through sexual contact with infected spouse, and intravenous drug abuse, were all associated with decreased chances of delayed or late presentation to care with most associations statistically significant (Table 3).

**Table 3:** Multinomial regression for association analysis of late presentation with patient factors

Patient Factor	Late Presenters		Very Late Presenters	
	
	OR (95% CI)	p value	OR (95% CI)	p value
**Sex**				

Male	Reference			

Female	0.49 (0.41-0.54)	**< 0.001**	0.21 (0.15-0.30)	**< 0.001**

**Age Group**				

0-14 years	0.33 (0.23-0.5)	**< 0.001**	0.23 (0.1-0.56)	**0.0005**

15-24 years	0.40 (0.30-0.50)	**< 0.001**	0.27 (0.15-0.5)	**< 0.001**

25-34 years	Reference			

35-44 years	1.47 (1.26-1.73)	**< 0.001**	2.34 (1.7-3.2)	**< 0.001**

45 years & above	1.26 (1.02-1.56)	**0.034**	1.9 (1.2-3.04)	**0.006**

**Transmission route**				

Heterosexual	Reference			

HIV+ spouse	0.32 (0.27-0.40)	**< 0.001**	0.10 (0.05-0.16)	**< 0.001**

Parent to child	0.21 (0.13-0.34)	**< 0.001**	0.10 (0.03-0.35)	**< 0.001**

Transfusion recipient	1.8 (1.4-2.3)	**< 0.001**	0.92 (0.6-1.4)	0.72

IVDU	0.49 (0.24-0.95)	**0.03**	0.26 (0.05-1.2)	0.06

Unsterile syringe use, Occupational exposure	2.16 (1.03-4.5)	**0.04**	2.3 (0.42-12.8)	0.32

MSM	3.4 (0.35-32.73)	0.26	3.5 (0.14-85.7)	0.28

Unknown, undisclosed	1.3 (1.1-1.54)	**0.003**	1.08 (0.77-1.5)	0.6

## Discussion

Late presentation with low baseline CD4 T cell counts and presence of AIDS defining and/or AIDS associated symptoms is the strongest prognostic marker for early mortality in both low and high income countries. Generally late testing and presentation to HIV care centres and programs is a result of diagnostic testing as a consequence of symptoms, while earlier diagnosis is more typical of patient self-request for testing, self-perceived risk, or universal screening [6]. An alarmingly high proportion of HIV infected individuals are reported to present late for care in developing countries like Sub-Saharan Africa, South-East Asia, South America etc due to limited health care access and health literacy [13,14]. Several studies have identified delayed presentation to care, rather than a late diagnosis, as cause of late initiation of HAART and thus an underlying factor for poor prognosis [15].

In the present retrospective study, we set out to examine the level of late presentation to care of HIV infected Indian individuals in a tertiary health care setting and to identify the extent of immunosuppression in these subjects along with presence of opportunistic infections. The study population was mostly male of the reproductive age group of 25-44 years thereby highlighting the most affected population. The significantly low number of females coming to the laboratory for CD4 T cell count underscores the vast gap in health care uptake between males and females. Heterosexual route of transmission was the most common transmission route reported by patients as a possible mode of disease acquisition as is true for majority of HIV cases reported throughout India.

Several previous studies have reported various ways of characterising late presenters to care. In our study, patients having AIDS associated symptoms like chronic diarrhoea, chronic fever, unexplained weight loss, oral ulcers, etc and AIDS defining illnesses like pulmonary and extra-pulmonary Tuberculosis (TB), STDs, Bacterial diarrhoea, *Pneumocystis carinii *pneumonia, along with patients having less than 200 CD4 T cells/uL of blood at the time of first CD4 T cell count, were classified as late presenters to care. We argued that the presence of chronic illnesses like fever and diarrhoea etc though not AIDS defining, are major pointers to raise suspicion of a possible HIV infection. Further such symptoms would highlight disease chronicity and thus should be labelled as a missed opportunity for early diagnosis and care. Nearly 80% of subjects were classified as late testers based on above criteria. This proportion is very high compared to other studies. As the setting for the study was one of the nation's largest tertiary health care facilities, majority of patients are referred here after efforts to treat them at primary and secondary health care levels and also at private settings. Therefore the proportion of sick and very sick patients is usually higher at the laboratory as compared to the clientele in private or secondary healthcare settings, and can partly explain the huge proportions of late presenters in our study. Also, our inclusion of chronic symptoms associated with HIV infection in addition to AIDS defining events and low CD4 T cell counts further increased this already large number of late presenters. A sizeable number of patients (15.4%) had CDC stage C HIV infection, ie clinical AIDS. This high number of AIDS cases at time of first presentation to care marks an alarming trend, clearly showing the lack of knowledge, and low risk perception of being possibly HIV infected and warrants further and renewed emphasis on health literacy.

Of all subjects studied, 14.4% had a pulmonary or extrapulmonary TB event at first time presentation to care. Management of TB in late presenters with CD4 T cell counts less than 200 cells/mL is a challenge due to an increased risk of developing IRIS and additive drug toxicities and interactions resulting from antimycobacterial therapy. Treatment in case of suspected IRIS involves close monitoring and symptomatic administration of steroids

At the time of first CD4 T cell count, 33% of subjects had a CD4 T cell count below 200 cells/uL with 9.5% subjects having CD4 T cell count below 50 cells/uL. This huge number of immunocompromised patients at first visit comprises subjects in need of immediate HAART initiation. Further when segregated on symptoms, 120 patients with CD4 T cell counts less that 200 cells/uL were found to be asymptomatic. As a sizeable part of the study population were ones who visited the laboratory before government of India roll out of free public sector ART and free CD4 testing facilities for HIV infected patients, many subjects without any apparent HIV related symptoms would have been missed for initiation of HAART. Further we observed in our study population, presence of patients with CD4 T cell counts well above 200 cells/uL and with chronic HIV associated symptoms without any AIDS defining illness. Treatment strategy for these patients is not clear in the currently available HIV-1 infection treatment guidelines, and thus most of them just get symptomatic treatment instead of initiation of HAART, thereby limiting the beneficial aspect of early HAART initiation. Men had a lower CD4 T cell count at first presentation to care for HAART evaluation as compared to females, which is consistent with previous study results. This can be attributed to the proportionately low utilization of health care facilities by men as compared to women.

In order to identify patient factors associated with late presentation to care, we performed multinomial logistic regression analysis to check the strength of association of each factor independently with late and very late presentation events. The factors associated with a risk of delayed presentation to care as uncovered in present study are consistent with results in other such studies [16-19]. Heterosexual males were more at risk of being late presenters as compared to females. The lower proportion of females being late presenters can be attributed to a higher uptake of Voluntary Counseling and Testing services by the females as part of routine health care services during pregnancy. Further females get tested soon after their spouse test HIV positive; thereby being tested at a much earlier stage of infection than their male counterparts, who generally get tested on developing symptoms. Also, self perception of illness and need to access health care is more in females as compared to males, which is highlighted in an almost double chance of late presentation in males [20,21]. Children diagnosed HIV positive had a minimal risk of late presentation. This is largely due to screening of pregnant females and provision of testing and treatment opportunities to children born to affected mothers.

Association of higher age groups of 35-44 years ages 45 years and above seen in our study with late and very late presentation to care has been emphasized in literature [19,22,23]. Homosexual transmission route has been reported to be associated with a lesser risk of late presentation in previous studies [23,24]. However, in our study we observed a very high risk of late presentation in this group though the association was not statistically significant. The lack of significance can be attributed to the very small number of subjects in this group. However, we would like to mention here that homosexuality was termed illegal in India till very recently and thus the stigmatization of homosexual relations is very high. This is a direct deterrent in utilization of HIV testing facilities and thus multiplies many folds the chances of delayed presentation. Injection drug users (IDUs) were another transmission group found be at a lower risk of late presentation in our data when compared to all other reported transmission routes. Previous studies have identified IDUs as a population at high risk of late diagnosis and care [23,25-27]. Identification of IDUs as a high risk population for HIV acquisition and various government and non-government bodies focusing on this population in terms of routine HIV testing and regular health check-ups could be the reason of lesser late presentation in these individuals as a group.

## Conclusion

In view of the results, there is a need for expanded routine testing of HIV infection with a stronger focus on men who are observed as most vulnerable population for late presentation. Also, emphasis should be given to testing for HIV in higher age group subjects as they are also more likely to be late presenters. The increased proportion of late presenters highlights the delay in treatment to these individuals, indicating the need to develop national level strategies effective in promoting early diagnosis and of HIV infection in order to increase the impact of universal access HAART. In view of the public health implication of early HIV diagnosis for reducing HIV transmission and preventing late presentation, HIV testing should be more frequently prescribed in all health care settings.

## Competing interests

The authors declare that they have no competing interests.

## Authors' contributions

KM and MV contributed to study inception. MV supervises the laboratory involved in HIV diagnosis and testing. KM, MV, NKC, and SM participated in data analysis, and interpretation. MV provided data analysis inputs. KM and MV drafted the manuscript. All authors read and approved the final manuscript. MV takes responsibility for the paper as a whole.

## Pre-publication history

The pre-publication history for this paper can be accessed here:

http://www.biomedcentral.com/1471-2458/10/416/prepub
